# The effects of intravenous lignocaine on depth of anaesthesia and intraoperative haemodynamics during open radical prostatectomy

**DOI:** 10.1186/s13104-017-2570-4

**Published:** 2017-07-06

**Authors:** Laurence Weinberg, Jae Jang, Clive Rachbuch, Chong Tan, Raymond Hu, Larry McNicol

**Affiliations:** 1 Department of Surgery, The University of Melbourne, Austin Health, VIC Australia; 2 Department of Anaesthesia, Austin Health, Heidelberg, VIC Australia; 3 Faculty of Medicine, The University of New South Wales, Sydney, Australia; 4 Department of Anaesthesia, Eastern Health, Box Hill, VIC Australia

**Keywords:** Lignocaine, Lidocaine, Volatile agents, Depth of anaesthesia, Sevoflurane, Haemodynamics, Blood pressure, Fluids

## Abstract

**Background:**

Lignocaine is a local anaesthetic agent, which is also commonly used as a perioperative analgesic adjunct to accelerate rehabilitation and enhance recovery after surgery. Lignocaine’s systemic effects on intraoperative haemodynamics and volatile anaesthetic requirements are not well explored. Therefore, we evaluated the effects of intravenous lignocaine on intraoperative volatile agent requirements and haemodynamics in patients undergoing major abdominal surgery.

**Methods:**

We performed an analysis of 76 participants who underwent elective open radical retropubic prostatectomy. Patients received lignocaine (1.5 mg/kg loading dose) followed by an infusion (1.5 mg/kg/h) for the duration of surgery, or saline at an equivalent rate. The aims of the study were to evaluate the end-tidal sevoflurane concentration required to maintain a bispectral index of between 40 and 60. Measurements included intraoperative blood pressure, heart rate, and the volume of intravenous fluids and dosage of vasoactive medications administered.

**Results:**

The average end-tidal sevoflurane concentration was lower in the Lignocaine group compared to saline [1.49% (SD: 0.32) vs. 1.89% (SD: 0.29); 95% CI 0.26–0.5, p < 0.001]. In the Lignocaine group, the average mean arterial pressure was 80.3 mmHg (SD: 4.9) compared to 85.1 mmHg (SD: 5.4) in the Saline group (95% CI 2.4–7.1, p < 0.001). Systolic blood pressure was also lower in the Lignocaine group: 121.7 mmHg (SD: 6.1) vs. 128.0 mmHg (SD: 6.4) in the Saline group; 95% CI 3.5–9.2, p < 0.001, as was the mean heart rate [Lignocaine group: 74.9 beats/min (SD: 1.8) vs. 81.5 beats/min (SD: 1.7) in the Saline group, 95% CI 4.1–9.1, p < 0.001]. Maintenance fluid requirements were higher in the Lignocaine group: 3281.1 mL (SD: 1094.6) vs. 2552.6 mL (SD: 1173.5) in the Saline group, 95% CI 206–1251, p = 0.007. There were no differences in the use of vasoactive drugs.

**Conclusions:**

Intravenous lignocaine reduces volatile anaesthetic requirements and lowers blood pressure and heart rate in patients undergoing open radical prostatectomy.

## Background

Lignocaine is a local anaesthetic agent commonly used as a perioperative analgesic adjunct to accelerate rehabilitation and enhance recovery after surgery. In a recent multicentre randomised control trial, we found that the use of intravenous (IV) lignocaine was associated with shorter postoperative hospital stay, reduced pain at rest and reduced 24-h postoperative morphine consumption [1]. However lignocaine’s effects on the intraoperative volatile agent requirements and patient haemodynamics have not been consistently characterized. Animal studies have shown that the use of IV lignocaine has been associated with a reduction in the minimum alveolar concentration (MAC) of volatile anaesthetic agents [2–5]. However, to date there is limited research evaluating the relationship between IV lignocaine and volatile anaesthetic requirements and intraoperative haemodynamics in patients undergoing major surgery [6–8]. Given this gap in knowledge, we evaluated the effects of lignocaine on the requirement of volatile anaesthetic agents and intraoperative haemodynamics in patients undergoing open radical retropubic prostatectomy.

## Methods

All patients that participated in the original trial by Weinberg et al. [1] who underwent open radical prostatectomy were included in this analysis. In the original multicentre, double-blinded, randomised control study, adult patients over the age of 18 years, and American Society of Anaesthesiologists (ASA) class of I to III were randomly assigned to receive either IV lignocaine (loading dose followed by infusion) or normal saline. The primary outcome of the original study was the length of postoperative hospital stay and secondary outcomes included postoperative pain, analgesia, side effects, and participant satisfaction. However, the effects of IV lignocaine on intraoperative volatile anaesthetic requirements and patient haemodynamics were not reported. The primary aim of this present study is to report the effects of intraoperative IV lignocaine on end-tidal sevoflurane (ET-Sevo) concentration required to maintain a bispectral index of between 40 and 60. In addition, the effects of intraoperative IV lignocaine on blood pressure, heart rate, and volume of fluids, and dosage of vasoactive medications administered are presented.

The original study [1] was approved by Human Research Ethics Units at Austin and Box Hill hospitals (Number: 2008/03180) and registered with the Australian New Zealand Clinical Trials Registry (Number: 12609001073291). All participants provided written consent for the primary study. The Ethics Committee approved the data collection of all the variables reported in this secondary analysis and participant consent was not obtained. For the original study [1], exclusion criteria included laparoscopic surgery, allergy to morphine, non-steroidal anti-inflammatory drugs, and local anaesthetic agents, cardiac conduction defect, use of class I anti-arrhythmic agents or amiodarone, history of seizures, epilepsy, or craniotomy within the last 5 years, myasthenia gravis, cognitive impairment or mental illness, opioid tolerant patients or significant hepatic or renal impairment.

### Standardisation of anaesthesia

All patients had a fasting period of 2 h for clear fluids and 6 h for a light meal. There was no preoperative fluid loading. Immediately prior to induction of anaesthesia, the Lignocaine group received IV lignocaine (1.5 mg/kg loading dose) over a 3-min period followed by a continuous intraoperative infusion (1.5 mg/kg/h). The control group received normal saline at an equal infusion rate. Anaesthesia was induced with IV fentanyl (3 µg/kg), propofol (1–3 mg/kg), and a non-depolarising neuromuscular blocker. Maintenance of anaesthesia was with Sevoflurane, in 50% oxygen-air balance to maintain a target bispectral index (Aspect Medical BIS^®^) of between 40 and 60. Intraoperative analgesia was standardised with a fentanyl infusion (2.5 µg/kg/h). Maintenance fluid therapy consisted of a balanced crystalloid (5 mL/kg/h). Additional crystalloid boluses and colloid intervention were administered at the discretion of the treating anaesthetist. Blood transfusion was in accordance with the current Australian patient blood management guidelines [9]. Electrolyte disturbances were managed as per standard medical practice. Core temperature of greater than 36.0 °C was maintained with warm fluids and a forced-air warming device. Intraoperatively, hypotension was treated with fluid therapy, IV metaraminol (250–500 µg) or ephedrine (5–10 mg). Infusion of lignocaine or saline was stopped at the end of the operation on the last surgical stitch.

#### Types of statistical analysis used

To analyse the continuous data, Student’s t test and Mann–Whitney U tests were performed. To compare means, standard two-sample t tests were used. For categorical data, Chi squared tests were used with the Newcombe–Wilson method to calculate the 95% confidence interval [10]. p values of <0.05 were considered to be of statistical significance.

## Results

Of the 86 participants who consented to participate in the original study [1], nine were planned to undergo laparoscopic prostatectomy and were excluded. In total, 76 patients were eligible. Thirty-eight were assigned to the Lignocaine group and 38 patients were assigned to the Saline group. One patient from the Lignocaine group had his operation cancelled due to anaphylaxis caused by cefazolin. Hence, he was excluded from the study. Thus in total, 75 patients met the inclusion criteria and completed the study. The baseline characteristics of the study participants are presented in Table 1.

**Table 1:** Baseline demographics

	Lignocaine (n = 37)	Saline (n = 38)	p value
Age (years)	61 (6.3)	60 (7.6)	0.38
Weight (kg)	85 (14.1)	83 (11.9)	0.43
Body mass index (kg/m^2^)	28 (5.05)	26 (3.53)	0.80
ASA class			
I	24 (65%)	26 (68%)	0.74
II	13 (35%)	12 (32%)	0.74
III	0	0	
Gleason scores	7 (0.86)	7 (0.62)	0.91
PSA (ng/mL)	8.7 (5.02)	7 (4.85)	0.54
Co-morbidities	15 (40.5%)	12 (31.6%)	0.42
Hypertension	10 (27.0%)	9 (23.7%)	0.74
Diabetes	1 (2.6%)	3 (7.9%)	0.32
Peripheral vascular disease	1 (2.6%)	0	0.31
Chronic obstructive pulmonary disease	1 (2.6%)	0	0.31
Ischaemic heart disease	0	0	
Renal impairment	0	0	
Duration of surgery	155.7 min	141.6 min	0.13

Values are mean (SD) and number (proportion)

**Fig. 1:**
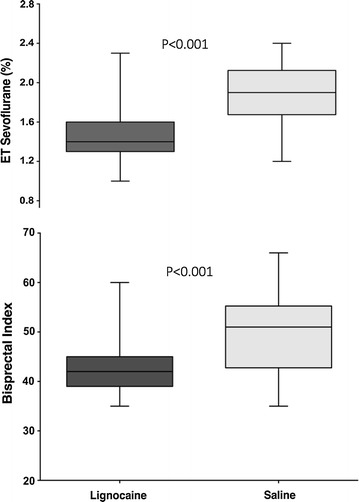
Box-and-whisker graph showing the end-tidal (ET) concentrations of sevoflurane and the intraoperative bispectral index values of patients receiving lignocaine or saline

**Fig. 2:**
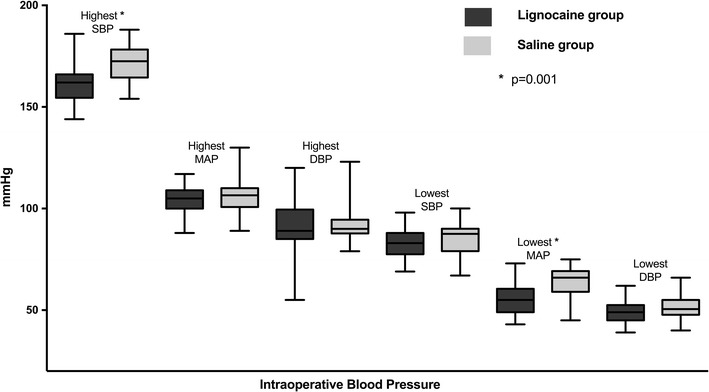
Box-and-whisker graph showing the highest and lowest systolic (SBP), mean (MAP) and diastolic (DBP) blood pressures during surgery

The ET-Sevo concentration to maintain a bispectral index of 40–60 was lower in the Lignocaine group compared to the Saline group [1.49% (SD: 0.32) vs. 1.89% (SD: 0.29); 95% CI 0.26 to 0.5, p < 0.001] (Fig. 1). The average bispectral index in the Lignocaine group was 43.4 (SD: 6.0) vs. 49.8 (SD: 8.2) in the Saline group (p < 0.001) (Fig. 1). The average blood pressures throughout surgery, together with highest, mean, and lowest heart rates are summarised in Table 2. The highest, mean, and lowest intraoperative blood pressures are presented in Fig. 2.

**Table 2:** Intraoperative haemodynamics

	Lignocaine (n = 37)	Saline (n = 38)	95% CI	p value
Average blood pressure (mmHg)				
Systolic	121.7 (6.1)	128.0 (6.4)	3.5 to 9.2	<0.001
Diastolic	70.2 (6.7)	72.1 (6.2)	−1.1 to 4.9	0.2
Mean	80.3 (4.9)	85.1 (5.4)	−1.1 to 4.9	0.2
Heart rate (beats/min)				
Highest	97.5 (7.1)	103.8 (9.7)	2.4 to 10.3	<0.001
Lowest	52.1 (7.7)	59.0 (7.4)	3.3 to 10.3	<0.001
Mean	74.9 (1.8)	81.5 (1.7)	4.1 to 9.1	<0.001
Number of patients requiring vasopressor use (ephedrine or metaraminol)	22 (59%)	15 (39%)	−26.5 to 42.6	0.1
Intraoperative temperature (°C)	35.5 (0.5)	35.6 (0.5)	−0.34 to 0.14	0.41
Average bispectral index (%)	43.4 (6.0)	49.8 (8.3)	0.34 to 0.14	0.13

Values are mean (SD) and number (proportion)

In total, the Lignocaine group required more crystalloids than those in the Saline group [3281.1 mL (SD: 1094.6) vs. 2552.6 mL (SD: 1173.5); p = 0.007]. Hartmann’s solution, which was the most used crystalloid, was administered in higher volumes in the Lignocaine group than in the saline group [3167.6 mL (SD: 1048.6) vs. 2281.6 mL (SD: 2281.6); p < 0.001). Gelofusine was the most used colloid and administered in similar amounts in both groups [530.6 mL (SD: 707.1) in the Lignocaine group vs. 500.0 mL (SD: 687.7); p = 0.6]. There were no significant differences in the amounts of albumen administered [112.9 mL (SD: 297.1) in the Lignocaine group vs. 126.3 mL (SD: 295.6); p < 0.65]. In the Lignocaine group, twenty-two patients (59%) required either intermittent boluses of either metaraminol or ephedrine compared to fifteen patients (39%) in the Saline group (p = 0.1). The median (IQR) dose of metaraminol in the Lignocaine group was 3 mg (2.8:4) vs. 3 mg (2:3) in the Saline group (p = 0.18). The median dose of ephedrine in the Lignocaine group was 15 mg (7.5:15) vs. 9 mg (6:10) in the Saline group (p = 0.27).

## Discussion

In this analysis of a multicentre, double-blinded, randomised control trial [1], the administration of a bolus dose of IV lignocaine, followed by an IV infusion was associated with a decreased requirement of volatile anaesthetic agents, as compared to saline. The ET-Sevo concentration required to maintain anaesthesia was reduced by 21%, and intraoperative systolic and mean arterial pressure and heart rate were significantly lower in the Lignocaine group. Whilst there is a growing body of evidence supporting the use of IV lignocaine in accelerating rehabilitation and improving outcomes after abdominal surgery [6–8], the present study shows that the intraoperative use of lignocaine also effects intraoperative haemodynamics and reduces the concentration of volatile agents required to maintain anaesthesia. These are important clinical implications for the optimal and safe provision of anaesthesia.

A recent systematic review (37 trials, 1429 patients) reported that IV lignocaine consistently attenuates cardiovascular response to laryngoscopy and tracheal intubation [11], however there are very few clinical studies that have methodically evaluated the intraoperative effects of systemically administered lignocaine on haemodynamics, volatile agent requirements, and the depth of anaesthesia. The use of IV lignocaine has been shown to be associated with a reduced requirement of intravenous anaesthetic agents, specifically propofol [12–15]. Intravenous lignocaine has also been shown to reduce the requirements of volatile anaesthetics in patients undergoing non-abdominal surgeries [16–18], findings similar to the present study. Interestingly, even the delivery of lignocaine via both the epidural and IV routes has been shown to reduce requirement of volatile anaesthetics [19, 20]. In laparoscopic abdominal surgery, IV lignocaine has reduced the average concentration of volatile anaesthetic agents by more than 35% [21, 22]. However, to our knowledge, in the context of major open abdominal surgery only two studies have evaluated the effects of IV lignocaine on the concentration of volatile anaesthetic required to maintain anaesthesia [16, 20]. Hamp et al. found that the mean alveolar concentration of sevoflurane was 12% lower in those receiving a bolus dose of 1.5 mg/kg of intravenous lignocaine [13, 16]. Kuo et al. administered 2 mg/kg bolus of IV lignocaine followed by an infusion of intravenous lignocaine (3 mg/kg/h), and reported that the mean ET-Sevo concentration was 18% lower in the Lignocaine group [20]. Unlike our study, neither of these trials reported any haemodynamic effects or cardiovascular changes with lignocaine. Furthermore, these studies are limited by the heterogeneity of the types of surgical procedures performed.

Paradoxically in our study, despite lower end-tidal concentrations of sevoflurane used to achieve 1 MAC of anaesthesia in the Lignocaine group, BIS values were significantly lower, a finding that reflects a greater depth of anaesthesia; this may explain the significantly lower blood pressures and heart rates observed in the Lignocaine group. In addition, this may further explain why participants in the Lignocaine group required higher volumes of intraoperative fluids, and why a greater proportion of participants required vasoactive medications to support blood pressure. Anaesthetists should be mindful of these haemodynamic effects, which are not commonly reported in many of the clinical studies evaluating lignocaine in the perioperative setting. Numerous trials have evaluated the effects of IV lignocaine on intraoperative haemodynamics and anaesthetic requirement [12–14, 16, 18, 20–26] (Table 3). Noteworthy, some of these studies were performed in small sample of patients [26–28], whilst others failed to investigate the effects of combining a loading dose of lignocaine with a continuous infusion [24, 27]. Our findings of lower end tidal concentrations of volatile agents in the Lignocaine group provide support for the inclusion of lignocaine in the combination of pharmacological agents that may contribute to “balanced anaesthesia”. Furthermore, it might also be feasible to use an infusion of lignocaine to attenuate the haemodynamic changes associated with open abdominal surgery.

**Table 3:** Literature review of the relevant trials evaluating the effects of intravenous lignocaine on intraoperative haemodynamics and anaesthetic requirements

Author	Patients (n)	Type of surgery	Lignocaine		Trial design	Variable measured	Outcome (compared to control)
			Bolus dose	Infusion dose			
Cassuto [25]	20	Elective Cholecystectomy	100 mg	2 mg/min/24 h	Single centre RCT	Intraoperative SBP	No significant differences
						Intraoperative HR	No significant differences
Rimback [26]	30	Cholecystectomy	100 mg	3 mg/min for 24 h	Single centre RCT	SBP during first postoperative day	No significant differences
						HR during first postoperative day	No significant differences
Ben-Shlomo [14]	90	Minor gynaecological surgery	3 mg/kg (intramuscular)	None	Double blinded factorial RCT	Total dose of propofol required to achieve loss in response	34.4% lower in the Lignocaine group (p < 0.001)
Waijma [23]	25	Electroconvulsive Therapy	1.5 mg/kg	None	Double blinded RCT	MAP before and during electroconvulsive therapy	No significant differences
						HR before and during electroconvulsive therapy	No significant differences
Kuo [20]	60	Elective colon surgery	2 mg/kg	3 mg/kg/h	Single centre, double blinded RCT	Mean end tidal desflurane concentration	18% lower in Lignocaine group. (p < 0.1)
Kaba [21]	40	Laparoscopic colectomy	1.5 mg/kg	2 mg/kg/h until end of operation, then 1.33 mg/k/g/h for next 24 h	Single centre, double blinded RCT	Intraoperative MAP	Lower in the Lignocaine group (p = 0.03)
						Intraoperative HR	Lower in the Lignocaine group (p = 0.002)
						Mean end tidal sevoflurane concentration	35% lower in the Lignocaine group (p < 0.001)
Saadawy [22]	120	Laparoscopic cholecystectomy	2 mg/kg	2 mg/kg/h for duration of surgery	Single centre, double blinded factorial RCT	MAP before induction, during operation, and in recovery	No significant differences
						HR before induction, during operation, and in recovery	No significant differences
						Mean end tidal sevoflurane concentration	48% lower in the Lignocaine group (p < 0.001)
Altermatt [12]	40	Elective laparoscopic cholecystectomy	1.5 mg/kg	2 mg/kg/h	Double blinded RCT	Mean maintenance propofol dose	17% lower in the Lignocaine group (p = 0.01)
Choi [18]	60	Breast plastic surgery	1.5 mg/kg	1.5 mg/kg/h	Single centre RCT	Mean intraoperative end tidal sevoflurane concentration	5% lower in the Lignocaine group (p = 0.014)
Hamp [16]	90	Elective surgery	1.5 mg/kg	None	Double blinded factorial RCT	Intraoperative mean alveolar concentration of sevoflurane	12% lower in the Lignocaine group (p = 0.022)
			0.75 mg/kg			Intraoperative mean alveolar concentration of sevoflurane	No significant differences when compared with the Saline group
Staikou [24]	78	Not specified	1.5 mg/kg	None	Double blinded RCT	SBP and DBP before induction and during surgery	No significant differences
						HR before induction and intraoperative	No significant differences
Weber [13]	54	Breast or orthopaedic surgery	1.5 mg/kg	None	Double blinded factorial RCT	Mean intraoperative plasma level of propofol required to prevent a movement response in 50% of patients (Cp50)	42% lower in the Lignocaine group (p < 0.05)
			0.5 mg/kg			Mean intraoperative Cp50 of propofol	No significant differences

There are several strengths to this study. First, we have investigated the combination of an IV loading dose and a continuous infusion of lignocaine on intraoperative haemodynamics and volumes of fluids used, as well as the concentration of volatile agent required to maintain anaesthesia. Second, we analysed data from a multi-centre study conducted in two teaching hospitals, providing some external validity and generalisability to other tertiary hospitals in developed countries. Third, the original trial was conducted under strict methodology, which minimises the risk of bias. Fourth, ex post facto calculation, when considering the concentration of volatile to maintain BIS value between 40 and 60, showed that a sample size of least 38 patients per group would be necessary to detect a clinically relevant 0.4% difference in ET-Sevo concentration, with a Type 1 error of 0.05 and a statistical power of 90%. The sample size in the present study is therefore completely consistent with this clinically important difference.

Our study however, does have several limitations. First, and most importantly our results may be considered less valid as the original trial [1], which was powered to evaluate length of hospital stay. Second, the original trial investigated generally healthy patients undergoing elective open radical prostatectomy. The results may not be applicable in patients with underlying comorbidities, including obesity or those undergoing emergency or other types of surgeries. Third, our study delivered a loading dose of 1.5/mg/kg of IV lignocaine followed by 1.5 mg/kg/h infusion for a 24-h period. Our results may not be replicated when different doses of administration are used. Fourth, our study recorded the average haemodynamics and the ET-Sevo concentration taken throughout the operation. Our results may not be extrapolated to compare the efficacy of adjuvant lignocaine to given stimuli at specific points in time e.g. endotracheal intubation or surgical incision.

## Conclusions

In conclusion, despite methodological limitations of the study, we have found that a loading dose of IV lignocaine followed by an intraoperative infusion significantly reduces the ET-Sevo concentration required to maintain anaesthesia during open radical prostatectomy. Further, this regimen was associated lower BIS values, and lower blood pressures and heart rates compared to saline. More participants receiving IV lignocaine required vasoactive therapy support, and a higher volume of IV fluid therapy was administered. Our results provide additional information regarding the potential contributions of parenteral lignocaine to modern balanced anaesthesia.

## Abbreviations

IV: intravenous
SD: standard deviation
BIS: bispectral index
CI: confidence interval
MAC: minimum alveolar concentration
ASA: American Society of Anaesthesiologists
ET-sevo: end-tidal sevoflurane
SBP: systolic blood pressure
MAP: mean arterial pressure
DBP: diastolic blood pressure
RCT: randomised control trial
PSA: prostate-specific antigen
HR: heart rate
